# Numerical simulation of spatiotemporal red blood cell aggregation under sinusoidal pulsatile flow

**DOI:** 10.1038/s41598-021-89286-1

**Published:** 2021-05-11

**Authors:** Cheong-Ah Lee, Dong-Guk Paeng

**Affiliations:** 1 Department of Ocean System Engineering, Jeju National University, Jeju, Korea; 2 Department of Radiology and Medical Imaging, University of Virginia, Charlottesville, VA USA

**Keywords:** Computational biophysics, Biophysics, Biomedical engineering, Fluid dynamics

## Abstract

Previous studies on red blood cell (RBC) aggregation have elucidated the inverse relationship between shear rate and RBC aggregation under Poiseuille flow. However, the local parabolic rouleaux pattern in the arterial flow observed in ultrasonic imaging cannot be explained by shear rate alone. A quantitative approach is required to analyze the spatiotemporal variation in arterial pulsatile flow and the resulting RBC aggregation. In this work, a 2D RBC model was used to simulate RBC motion driven by interactional and hydrodynamic forces based on the depletion theory of the RBC mechanism. We focused on the interaction between the spatial distribution of shear rate and the dynamic motion of RBC aggregation under sinusoidal pulsatile flow. We introduced two components of shear rate, namely, the radial and axial shear rates, to understand the effect of sinusoidal pulsatile flow on RBC aggregation. The simulation results demonstrated that specific ranges of the axial shear rate and its ratio with radial shear rate strongly affected local RBC aggregation and parabolic rouleaux formation. These findings are important, as they indicate that the spatiotemporal variation in shear rate has a crucial role in the aggregate formation and local parabolic rouleaux under pulsatile flow.

## Introduction

Cardiovascular disease is one of the most common causes of death worldwide^1^. Abnormally high range of red blood cell (RBC) aggregation is measured in patients with critical circulatory diseases of various organs, including myocardial ischemia and heart disease^2–8^. The rheological properties of RBC aggregation have become an important indicator for diagnosing some cardiovascular diseases^9–11^. There is rich literature dealing with RBC aggregation for a variety of clinical and pathological implications^8,12–14^. Recently, it has been reported that quantitative measurement of RBC aggregation could be applied as an inflammatory blood marker in critical situations^15^. Thus, it is obviously important to understand the behavior of RBC aggregation. RBC aggregation is a dominant factor in the rheological properties of blood. Previous studies have suggested that RBC aggregation is dependent on the hydrodynamic characteristics of flow^16–18^. Early experimental and computational studies have demonstrated that both RBC aggregation and viscosity are inversely related to the shear rate under steady Poiseuille flow^19–21^. RBCs strongly form a rouleaux formation at a low shear rate. With increasing shear rate, the RBC aggregation becomes progressively dissociated. Under steady Couette and Poiseuille flow, the RBC aggregates is observed to be in a linearly stacked formation, similar to coins. In contrast to steady Poiseuille flow, oscillatory flow in the arteries near the heart periodically generates a spatial variation in velocity distribution due to pressure changes during a cycle. Blood flow is affected by the morphological and physical properties of the vessel and the elasticity of the wall^22^. Therefore, understanding the behavior of RBCs while considering the hydrodynamic characteristics and geometrical properties under pulsatile flow is important.

The mechanism of RBC aggregation has not been sufficiently elucidated, and there are at least two models for the mechanism of RBC aggregation (i.e., depletion and bridging models). The depletion model of RBC aggregation explains that macromolecule exclusion near cell surfaces leads to osmotic gradients, depletion interactions, and attractive forces^23,24^. Based on deletion theory, the dynamic motion of RBC can be modeled to simulate the interaction energy among RBCs in polymer solutions^23,25–28^. Through numerical simulation with fluid-cell and cell-to-cell interactions, RBCs adhere and dissociate in the micro stenosis channel^29^. Aggregated RBCs are dependent on the dissociation of the cell membrane, intercellular strength, and fluid conditions. In addition, traveling RBCs were dissociated and transiently distributed as a parabolic shape through the stenosis channel. In our previous simulation work, the dynamic process of RBC aggregation repeatedly formed a parabolic shape that broke under sinusoidal pulsatile flow^26^. RBC particles were simply driven by Newton's second law with elastic, aggregational, and hydrodynamic forces under sinusoidal pulsatile flow. The formation of parabolic RBC aggregation was clearly observed if the amplitude of velocity was increased or the mean flow velocity was decreased. However, previous studies qualitatively showed the tendency of parabolic rouleaux, which was dependent on the amplitude and mean of the sinusoidal pulsatile flow velocity, but these studies did not provide the appropriate analysis of the mechanism. Therefore, it is necessary to analyze what hemorheological factors cause parabolic RBC aggregation under pulsatile flow. It is important to point out that pulsatile flow is a dynamic process in time and space, with axial and radial variations in flow, and that it results in RBC aggregation, in contrast to static-steady flow. In this work, we quantitatively investigated the relationship between the spatiotemporal variation in sinusoidal pulsatile flow and the local distribution of parabolic RBC aggregation.

This paper focuses on the introducing of a new hydrodynamic factor to interpret the distribution of local RBC aggregation under sinusoidal pulsatile flow. Specifically, we decomposed the shear rate into axial and radial components to investigate the effects of the spatial variation in the shear rate on RBC aggregation. The formation of local parabolic rouleaux cannot be explained by the radial shear rate of flow alone but rather should be associated with the radial and axial variations under pulsatile flow. Sinusoidal pulsatile flow was considered to emphasize the influence of axial gradient of flow velocity on the local parabolic shape of RBC rouleaux. This sinusoidal pulsatile flow is not realistic but artificial. However, this analysis of local RBC aggregation with axial and radial variations of flow velocity can be applied to any flow conditions where there is the axial velocity gradient to a certain level. This approach has not been reported in other studies because no or slight axial variation in shear rate has been observed under steady and pulsatile flow, respectively. To investigate the effect of two components of shear rate on RBC aggregation, we quantified the number of aggregated RBCs as a function of the radial and axial components of shear rate in a region of interest (ROI). Shear rates from the ROIs could be quantified and correlated with the distribution of RBC aggregation. The results demonstrated that a specific range of the axial component of shear rate and its ratio with the radial component of shear rate affected RBC aggregation and the local formation of a parabolic rouleaux shape. Hydrodynamic parameters (i.e., amplitude and mean flow velocity of sinusoidal flow) with the corresponding shear rate in these two components can determine the rouleaux size and local aggregation of RBCs. These findings suggest that the spatiotemporal variation in the two components of shear rate is an important factor affecting the aggregate formation and local parabolic rouleaux under pulsatile flow.

## Theory and results

**Figure 1:**
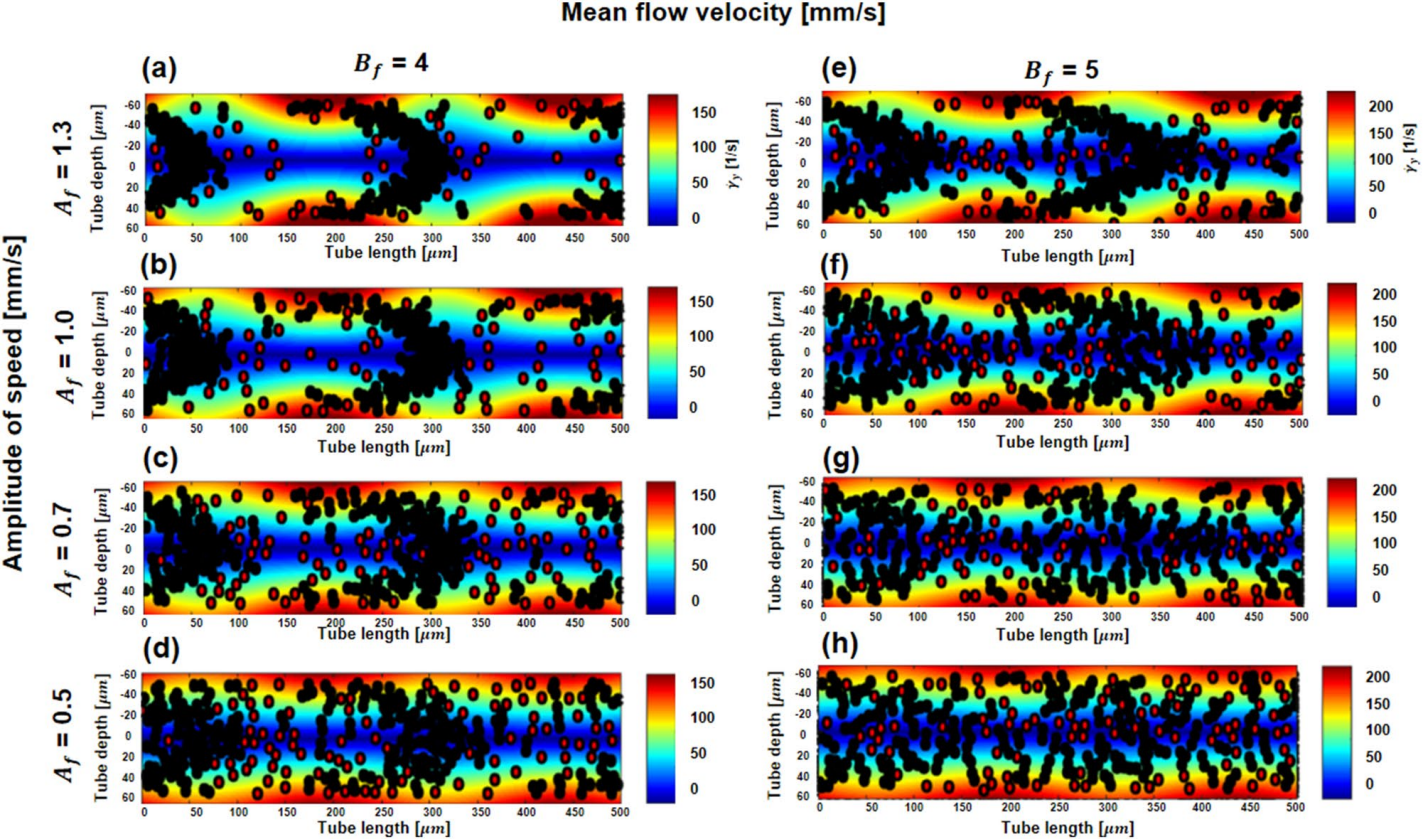
Snapshot of the distribution of RBCs showing formation and destruction of parabolic RBC rouleaux under sinusoidal pulsatile flow with different flow conditions at 0.96 s. Black and red particles represent aggregated and nonaggregated individual RBCs, respectively. Color bar is shear rate [1/s]. (**a–d**) RBC and its rouleaux distribution at different velocity amplitudes from 0.5 to 1.3 mm/s at a mean flow velocity of 4 mm/s. (**e**–**h**). The same amplitudes of flow velocity at a higher mean flow velocity of 5 mm/s. The lower the mean flow and the higher the velocity amplitude are, the stronger the local parabolic rouleaux.

The distributions of RBCs corresponding to the shear rate fields driven by pulsatile flow were dependent on the amplitude of velocity variation from 0.5 to 1.3 mm/s at one-time point of a cycle (Fig. 1a–d). The parabolic shape of aggregated RBCs (Fig. 1e–h) became less common when the mean flow velocity increased from 4 to 5 mm/s. Local distribution of aggregated RBCs was clearly observed when the mean flow velocity was low and the velocity amplitude was high. The results are similar to our previous study for RBC aggregation; the RBC aggregation level is dependent on hydrodynamic conditions^26^. The hydrodynamic parameters describing the spatial variation in fluid properties, such as velocity amplitude and mean flow velocity, affected the shear rate. During a cycle under sinusoidal pulsatile flow in the rigid tube, RBC aggregates repeatedly formed a local parabolic RBC rouleaux and then dissociated the parabolic shape. To show the cyclic variation of the formation of RBC aggregation, we provided a video file to show RBC motion corresponding to the velocity field in the supplementary material (see Supplementary video S1 online). In the numerical simulation, the sinusoidal pulsatile flow was the combination of Poiseuille flow in the y-direction (radial direction) and sinusoidal flow traveling at a certain mean flow velocity along the x-axis (axial direction). The sinusoidal pulsatile flow is defined in the following Equation:

1 $$u\left(t,x,y\right)={\{A}_{f}\mathrm{sin}\left(\omega t-{k}_{f}{x}\right)+{B}_{f}\}\left({1-\frac{4}{{h}^{2}}y}^{2}\right)$$

where ${A}_{f}$ and ${B}_{f}$ are the amplitude of the flow velocity and mean flow velocity, respectively. ${k}_{f}$ is the wavenumber, $\omega$ represents the wave angular frequency (2π*f*) propagating along the *x*-axis during a cycle corresponding to a frequency (*f*) of 1 Hz (= 60 BPM), and *h* is the tube diameter. For sinusoidal pulsatile flow, the velocity changes in two directions: the axial direction ($\mathrm{x}$), as flow propagation, and the radial direction ($\mathrm{y}$), as a parabolic profile. Therefore, two components of the flow velocity gradient for the sinusoidal pulsatile flow are as follows:

2 $$ \nabla u\left( {t,x,y} \right) = \frac{\partial u}{{\partial x}}\vec{x} + \frac{\partial u}{{\partial y}}\vec{y} = {\dot{\gamma }}_{x} \vec{x} + {\dot{\gamma }}_{y} \vec{y} $$

3 $${\dot{\upgamma }}_{y}\left(t,x,y\right)={\frac{\partial u}{\partial y} =-\{A}_{f}\mathrm{sin}\left(\omega t-{k}_{f}x\right)+{B}_{f}\}\left(\frac{8y}{{h}^{2}}\right)$$

4 $${\dot{\upgamma }}_{x}\left(t,x,y\right)={\frac{\partial u}{\partial x} =\{-A}_{f}{k}_{f}\mathrm{cos}\left(\omega t-{k}_{f}x\right)\}\left({1-\frac{4}{{h}^{2}}y}^{2}\right)$$

In Eq. (2), the spatial gradient of the flow velocity is composed of two components, the radial (${\dot{\upgamma }}_{y}$) and axial (${\dot{\upgamma }}_{x}$) shear rates, as shown in Eqs. (3) and (4), respectively. The shear rate has an amplitude of $\dot{\upgamma }=\sqrt{{({\dot{\upgamma }}_{y})}^{2}+{({\dot{\upgamma }}_{x})}^{2}}$ and direction of φ = tan^−1^(${\dot{\upgamma }}_{y}$/${\dot{\upgamma }}_{x}$). The shear rate in the y-direction is usually considered because a velocity change in the x-direction is absent for Poiseuille flow. However, the shear rate in the x-direction should be counted for sinusoidal pulsatile flow in Eq. (4).

**Figure 2:**
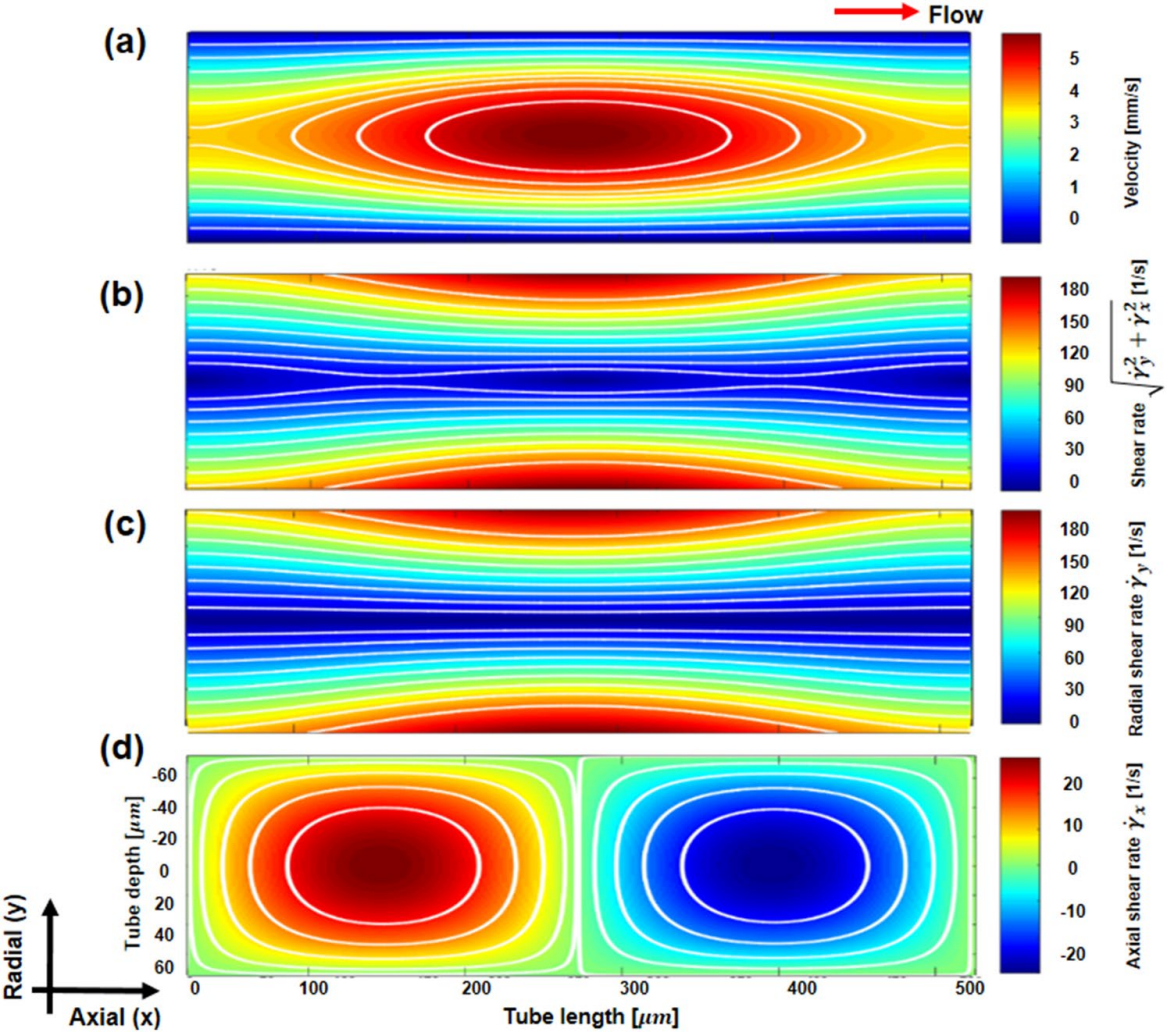
(**a**) Flow velocity field under the sinusoidal pulsatile flow with 4 mm/s of mean flow velocity and 1.0 $\mathrm{mm}/\mathrm{s}$ of velocity amplitude variation. (**b**) Shear rate field combined with radial shear rate and axial shear rate. (**c**) Radial shear rate (${\dot{\upgamma }}_{y}$) field derived by the spatial gradient field of velocity in the radial direction. (**d**) Axial shear rate (${\dot{\upgamma }}_{x}$) field derived by the spatial gradient of velocity in the axial direction. The color scales represent flow velocity ($\mathrm{mm}/\mathrm{s})$, shear rate [${s}^{-1}$], and radial and axial shear rates [${s}^{-1}$].

The ratio of axial and radial shear rates (${\dot{\upgamma }}_{x}/{\dot{\upgamma }}_{y}$) at the central area of the tube changed spatiotemporally with the flow conditions. When this ratio increased, the influence of the axial shear rate on RBC aggregation was relatively important to form a local parabolic of rouleaux under sinusoidal pulsatile flow. The flow velocity field and shear rates are shown in Fig. 2 at a specific time (t/T = 0), with a velocity amplitude of 1 mm/s and a mean flow velocity of 4 mm/s. In this figure, the white solid lines indicate 10 divided sections of flow velocity and shear rates with the same area. The shear rate is combined with radial and axial components (Fig. 2b). The decomposed shear rate fields show the radial shear rate (${\dot{\upgamma }}_{x}$) (Fig. 2c) and the axial shear rate (${\dot{\upgamma }}_{x}$) (Fig. 2d). The radial shear rate range is approximately 0–200 s^−1^, and the axial shear rate range is approximately − 25 s^−1^ to 25 s^−1^. The shear rate near the center was mainly affected by the axial shear rate due to the low mean radial shear rate of approximately 20 s^−1^, while the shear rate was generally similar to the radial shear rate except in the central area, as shown in Fig. 2b,c. RBC aggregation distributions considering the spatial variation in the shear rate fields were confirmed by increasing the amplitude of velocity from 0.5 to 1.3 $\mathrm{mm}/\mathrm{s}$ with a mean flow velocity of 4 $\mathrm{mm}/\mathrm{s}$.

**Figure 3:**
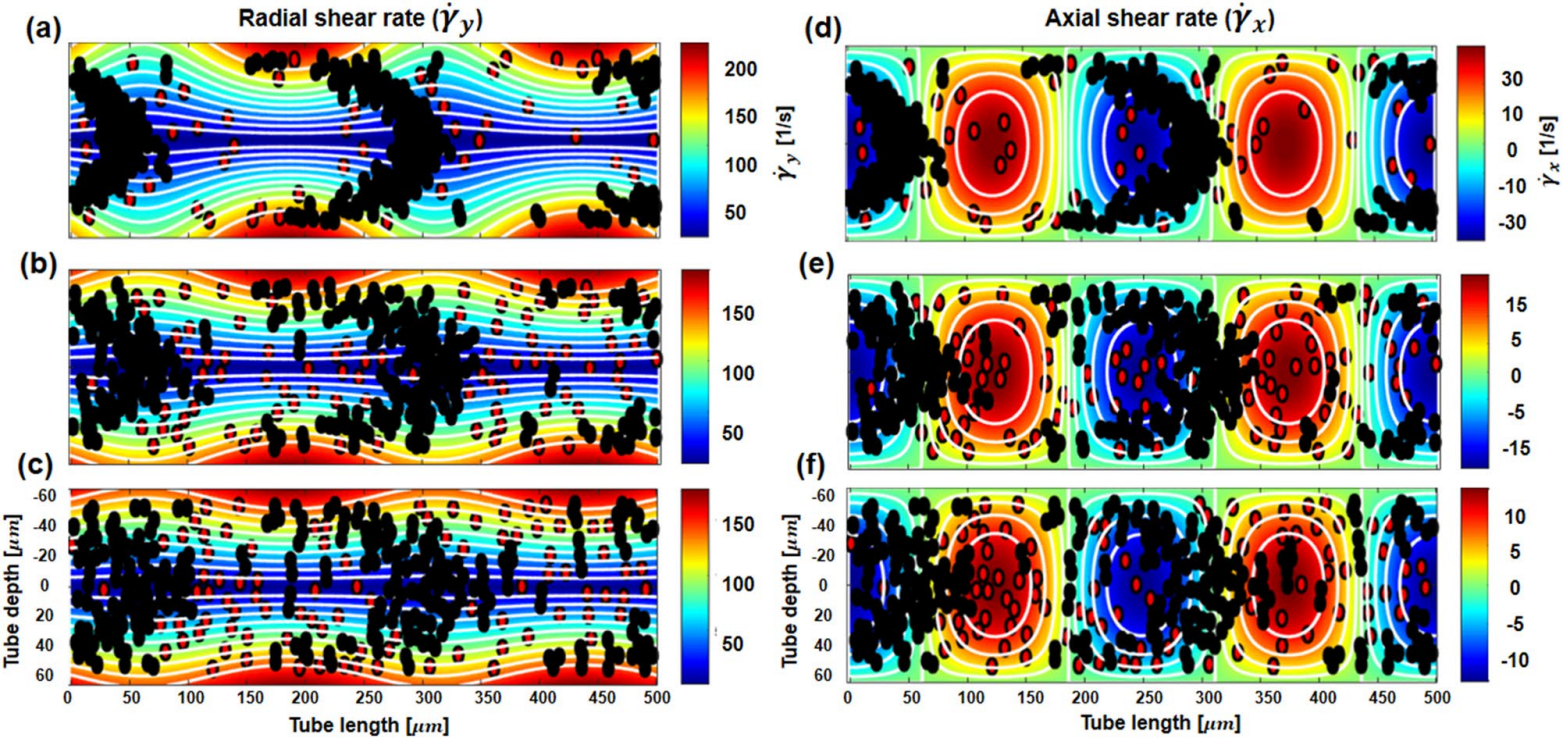
2D simulation results of RBC motion under sinusoidal pulsatile flow, corresponding to the spatial variation in two different components of shear rate. White lines represent the same shear rate range with equal area divided into 10 intervals. The RBC kinetics between hydrodynamic properties and local RBC aggregation were observed by decreasing the amplitude of velocity from 1.3 to 1.0 and 0.5 mm/s with a mean flow velocity of 4 mm/s (shown from top to bottom). When the velocity amplitude decreased, the maximum ranges of the radial and axial shear rates also decreased. (**a**–**c**) and (**d**–**f**) are the distribution of RBC aggregation coincided with the radial and axial shear rate fields, respectively. The spatial variation in axial shear rate is obviously related to the parabolic rouleaux distribution and local hematocrit, following the contour lines from the minimum to increasing axial shear rate. A supplementary video file is provided to show the dynamic RBC motion with shear rate fields for (**a**) and (**d**) (Supplementary video S1).

We attempted to explain RBC aggregation and parabolic rouleaux as a function of the radial shear rate (Fig. 3a–c) and axial shear rate (Fig. 3d–f) in the ROIs. The white solid lines represent 10 ROIs of the same area and the same shear rate range. Increasing the amplitude of velocity significantly changes the spatial distribution of the radial shear rate fields (Fig. 3a–c). The distribution of RBCs did not follow the radial shear rate field. The axial shear rate fields and RBC distributions are shown in Fig. 3d–f. The distribution of RBC aggregation was obviously related to the axial shear rate. Local aggregation occurred at a specific axial shear rate, and the parabolic rouleaux shape was clearly observed with increasing axial shear rate from the minimum value.

**Figure 4:**
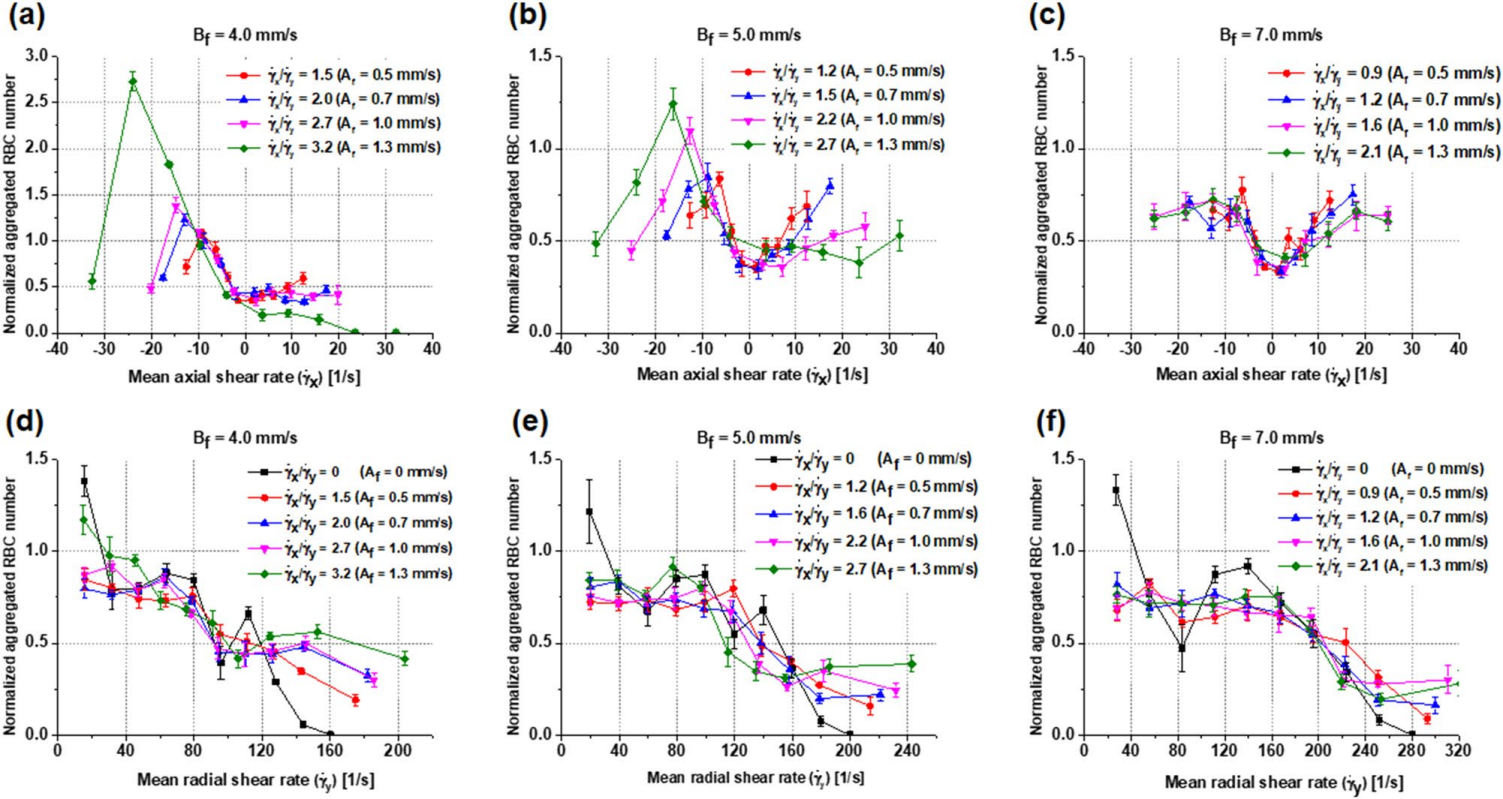
Normalized aggregated RBC numbers were computed as a function of the axial and radial shear rates via the ratio of axial shear rate (${\dot{\upgamma }}_{x}$) over radial shear rate (${\dot{\upgamma }}_{y}$) at the tube center, with changing velocity amplitude from 0 to 1.3 mm/s and mean flow velocity of 4, 5, and 7 mm/s at a normalized time (t/T = 0.9). Symbols and error bars indicate means and standard deviations over five periods, respectively. (**a**–**c**) and (**d**–**f**) are the normalized number of aggregated RBCs as a function of the mean axial shear rate (${\dot{\upgamma }}_{x})$ and mean radial shear rate (${\dot{\upgamma }}_{y}$) at 10 ROIs of the same area, respectively, for different amplitudes and means of flow velocity.

We compared the RBC distribution as a function of two components of shear rate in an ROI. The ratio of shear rates at the tube center area was changed to affect the RBC distribution with changing velocity amplitude from 0.5 to 1.3 mm/s with mean flow velocity of 4, 5, or 7 mm/s. Figure 4a–c and d–f show normalized aggregated RBC numbers as a function of axial mean shear rate (${\dot{\upgamma }}_{x}$) and radial mean shear rate (${\dot{\upgamma }}_{y}$), respectively. The number of aggregated RBCs was normalized by the number of randomly distributed RBCs in the ROI at the initial condition. The normalized aggregated RBC number is greater than 1 when the local hematocrit is higher than 40% in the ROI, mainly due to rouleaux formation. The maximum normalized number of RBC aggregates was approximately 2.7 at the axial shear rate of − 23 s^−1^ and the maximum shear rate ratio of 0.32 (A_f_ = 1.3 mm/s, and B_f_ = 4 mm/s) (Fig. 4a). When the maximum shear rate ratio decreased, the maximum normalized number of aggregated RBCs also decreased. The number of aggregated RBCs was also reduced significantly when the mean flow velocity increased from 4 to 7 mm/s (Fig. 4a–c), showing a minimal variation for a mean flow velocity of 7 mm/s. These results explain the effects of axial shear rate on RBC aggregation in terms of the rouleaux distribution and the local hematocrit in the specific ROI. When the shear rate ratio increased, the influence of the axial shear rate on RBC aggregation was relatively important to form a local parabolic shape of rouleaux under sinusoidal pulsatile flow. The normalized number of aggregated RBCs is shown as a function of the radial shear rate in Fig. 4d–f. For steady Poiseuille flow (solid black lines, shear rate ratio is 0), the normalized number of aggregated RBCs apparently decreases with increasing shear rate for all mean flow velocity from 4 to 7 mm/s. Near the tube walls under high shear rates, the normalized number of aggregated RBCs decreased to almost zero. The normalized RBC number was lower than 1 and did not change considerably under axial shear rates of 80, 100, and 160 s^−1^ for mean velocity of 4, 5, and 7 mm/s, respectively, with the exception of the shear rate ratio of 3.2.

**Figure 5:**
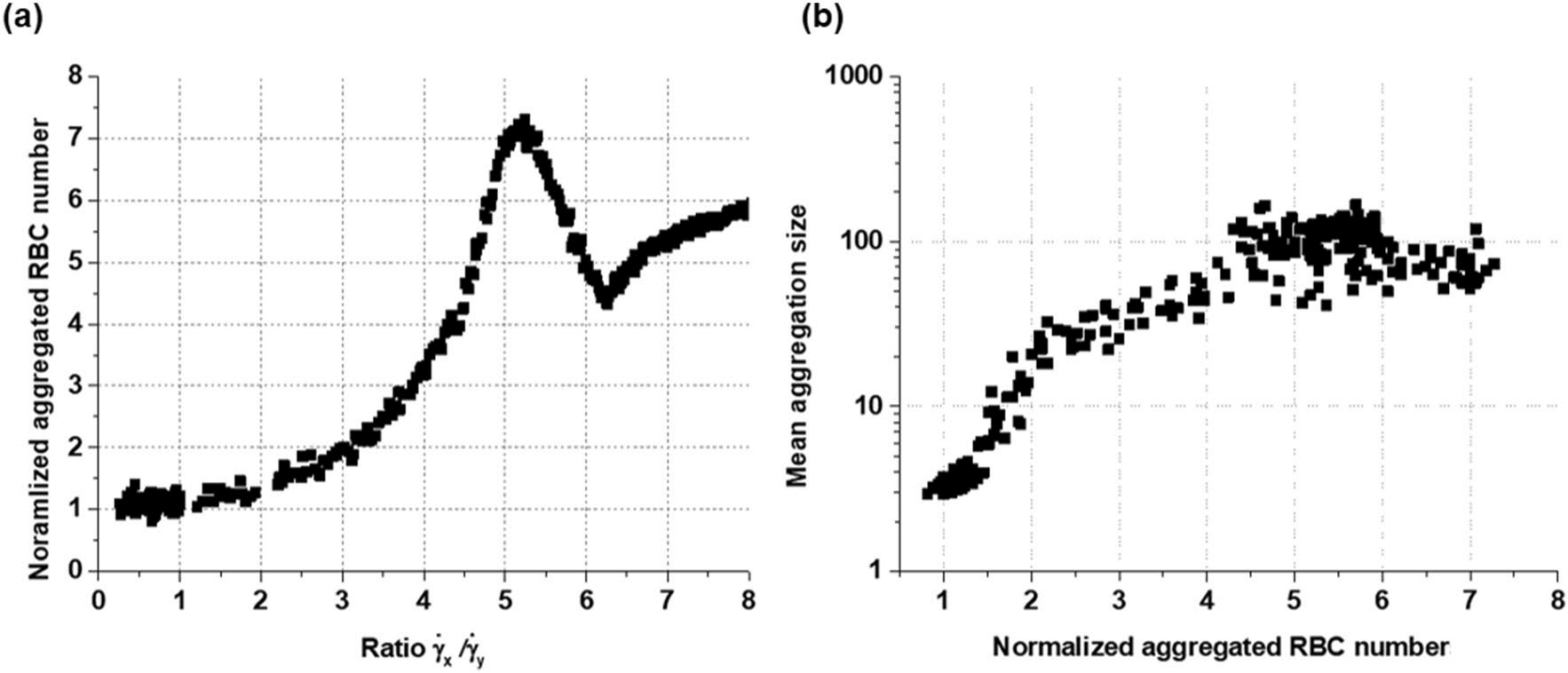
(**a**) Normalized aggregated RBC number as a function of the shear rate ratio between the axial and radial shear rates at the tube center area. Data were obtained from 300 different amplitudes of velocity from 0.1 to 5 mm/s with a mean flow velocity of 5 mm/s. (**b**) Mean aggregation size in a log scale as a function of the normalized aggregated RBC numbers.

Figure 5 shows the relationship between the number of normalized aggregated RBCs obtained from the peak aggregated RBCs in a specific ROI in the axial shear rate field and the ratio of axial to radial shear rates at the tube center. In these results, three hundred data points were obtained upon increasing the amplitude of velocity from 0.1 to 5 mm/s with a fixed mean flow velocity of 5 mm/s (Fig. 5a). An exponential increase in the number of normalized aggregated RBCs with shear rate ratio at the tube center was apparent from shear rate ratios of 0.1 to approximately 5.2, reaching a normalized aggregated RBC number of 7.5, mainly due to RBC aggregation and parabolic rouleaux formation. The maximum normalized number at the ratio of 5.2 is due to the tight parabolic rouleaux formation in a contour region at the ratio shown in Fig. 3d. Then, the normalized number decreased to 4.3 at the shear rate ratio of 6.5 before increasing and approaching 6 with an increasing shear rate ratio. The tight parabolic rouleaux shape started to diverge from the contour region to reach a local minimum at a ratio of 6.5 and converged to the contour region at a high ratio. The normalized number of aggregated RBCs is also decided by the aggregate size and local hematocrit; thus, the relationship between normalized aggregated RBC number and mean aggregate size is plotted in Fig. 5b. The mean aggregation size increased on a log scale with an increasing normalized number of aggregated RBCs and converged to approximately 100 (Fig. 5b).

**Figure 6:**
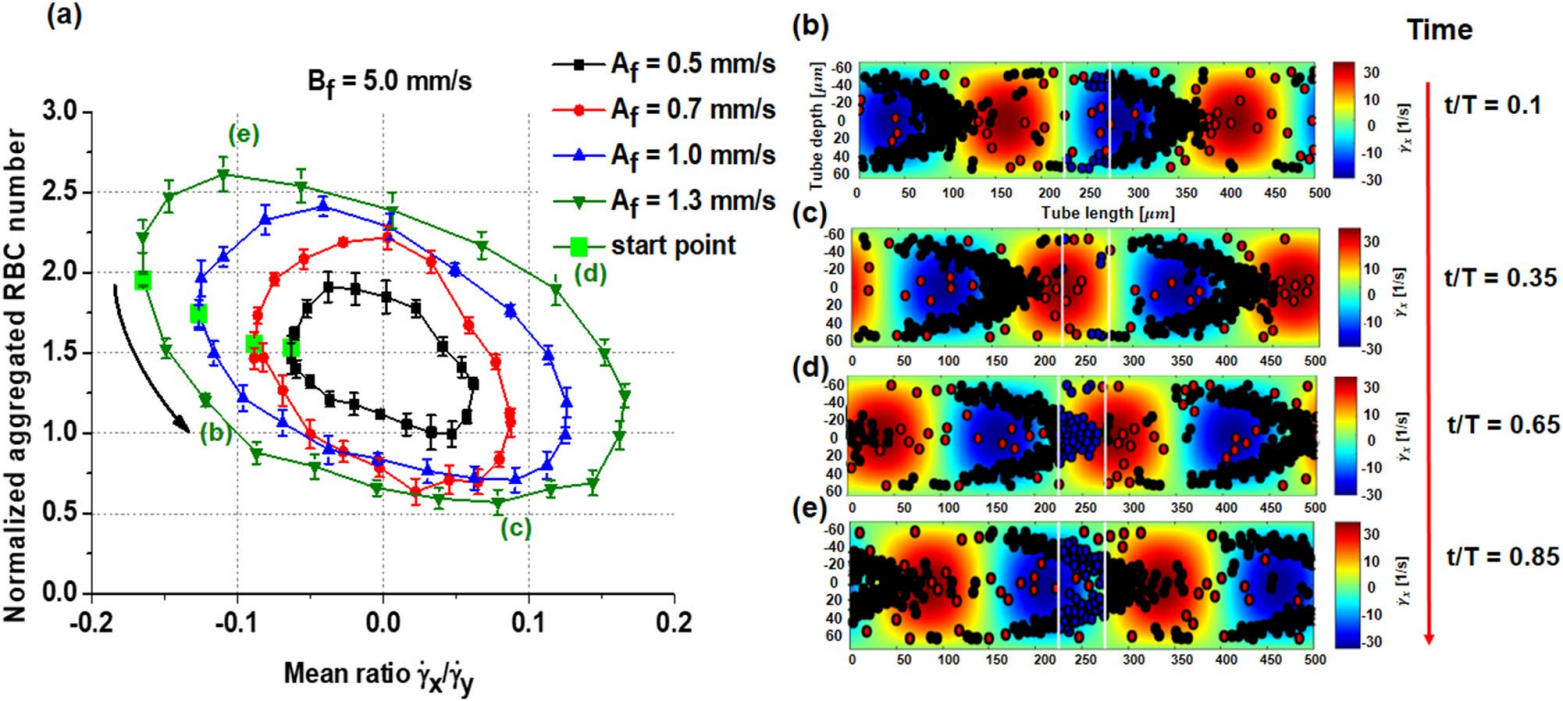
RBC aggregation with mean ratio of axial shear rate (${\dot{\upgamma }}_{x}$) to radial shear rate (${\dot{\upgamma }}_{y}$) in the rectangular ROI (0.1 $\times$ 0.1 ${\mathrm{mm}}^{2}$, white box near the tube center in the right panels) and its distribution with time over a pulsatile cycle. (**a**) With four different amplitudes of velocity, the normalized numbers of aggregated RBCs are shown as a function of mean shear rate ratio starting from the start points (green markers) in a counterclockwise direction during a cycle. The symbols and error bars are means and standard deviations over five cycles. (**b**–**e**) Corresponding RBC distributions overlapped with the axial shear rate at the normalized times of 0.1, 0.35, 0.65, and 0.85 in a period. The moments are marked in the left figures with a green solid line (${\mathrm{A}}_{f}=1.3 \mathrm{mm}/\mathrm{s}$). The aggregated RBCs (blue particles) were normalized over RBCs randomly distributed at the initial condition in the rectangular ROI.

**Figure 7:**
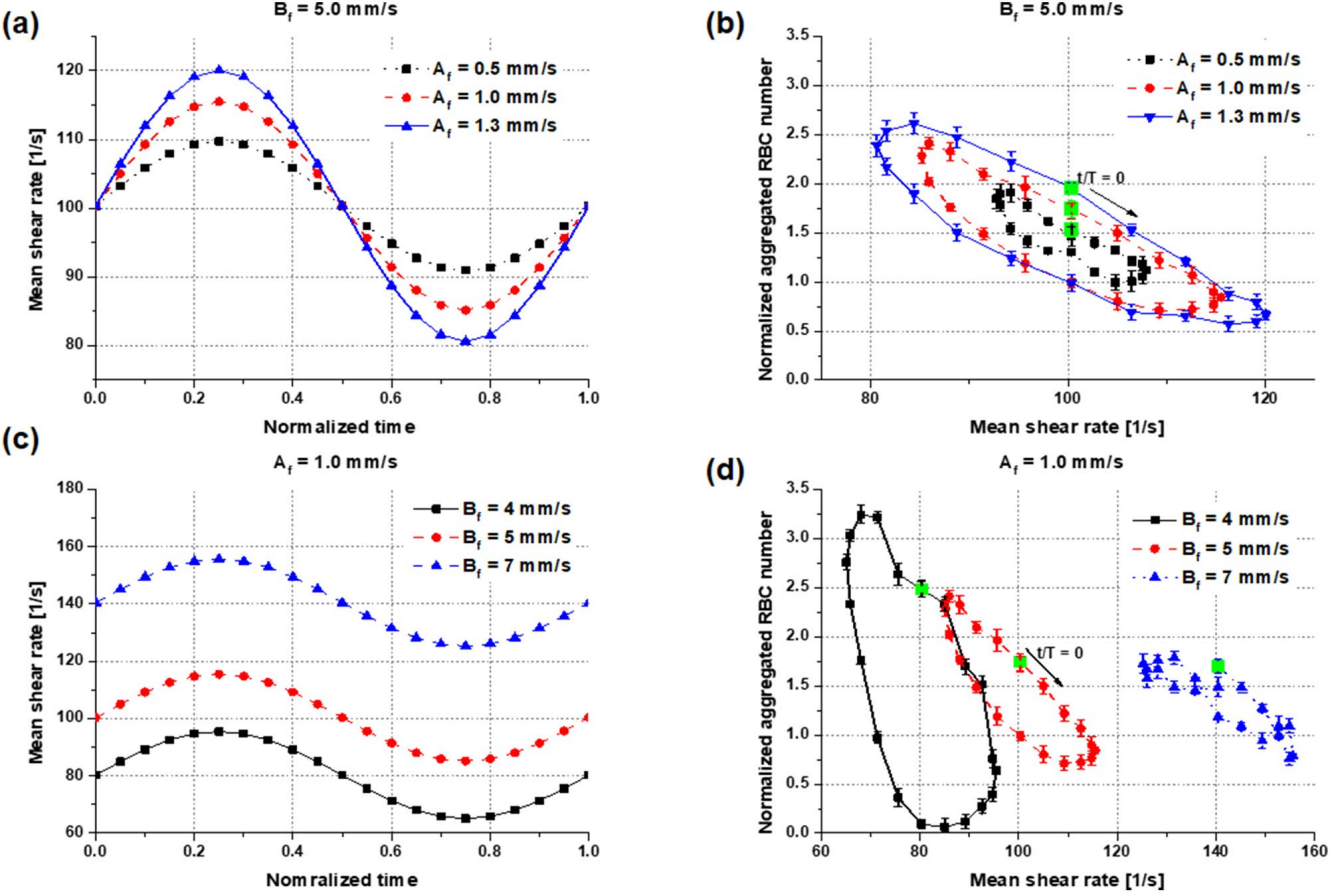
Mean shear rate and normalized aggregated RBC numbers in an ROI ($0.1\mathrm{ mm}$ of both tube diameter and length near the tube center). (**a**) Mean shear rate with normalized time over a cyclic period for three different amplitudes of velocity (0.5, 1.0, and 1.3 mm/s) with a fixed mean flow velocity of 5 mm/s. (**b**) Normalized aggregated RBC numbers averaged over five pulsatile cycles as a function of mean shear rate at three different hydrodynamic conditions. (**c**) and (**d**) are the same with (**a**) and (**b**), respectively, but for the different mean flow velocity from 4 to 7 mm/s with a fixed velocity amplitude of 1 mm/s. Symbols and error bars indicate the mean values and standard deviations over five cycles, respectively. From the start points (green symbols; t/T = 0), hysteresis loops show clockwise direction.

The normalized number of aggregated RBCs averaged over five cycles is plotted as a function of the mean shear ratio at the ROI (Fig. 6a). From the start points (green markers), the normalized numbers decreased when the mean shear ratio increased, and vice versa, but following counterclockwise hysteresis curves during a cycle. The variations in the normalized number and the mean shear ratio were larger for high-velocity amplitudes. The corresponding RBC distributions following a hysteresis loop for a velocity amplitude of 1.3 mm/s overlap with the axial shear rate field at normalized times of 0.1, 0.35, 0.65 and 0.85 (Fig. 6b–e). Tight parabolic rouleaux is formed in the region where the axial shear rate increases from the minimum, and the normalized aggregated RBC number is relatively small in the ROI where the axial shear rate is negative, as shown in Fig. 6a. The normalized number decreases where the axial shear rate is positive in the ROI, as shown in Fig. 6b. The local parabolic rouleaux is moved within the ROI where the mean ratio is positive, as shown in Fig. 6d. Then, the normalized aggregated RBC number peaks in the ROI where the ratio is negative, as shown in Fig. 6e. There, the local parabolic shape starts to break at the tube’ center area as it passes by the ROI. The normalized number of aggregated RBCs was averaged over five periods of sinusoidal pulsatile flow in an ROI (0.1 mm of both tube diameter and length) without considering spatial distribution. For three amplitudes of velocity (0.5, 1.0, and 1.3 mm/s), with a fixed mean flow velocity of 5 mm/s, the mean shear rates are shown with time over a cyclic period (Fig. 7a), and the normalized aggregated RBC numbers are plotted as a function of the corresponding mean shear rates (Fig. 7b) in the ROI. The normalized number of aggregated RBCs reduced when the shear rate increased and reached the minimum at the highest shear rate. Then, the normalized number of aggregated RBCs increased when the shear rate decreased, but to considerably lower numbers than those when the shear rate increased, resulting in a hysteresis curve during a cycle. The trends were more pronounced for high amplitudes of velocity. Then, the mean flow velocity was changed from 4 to 7 mm/s, with a fixed velocity amplitude of 1 mm/s; the resulting mean shear rates and normalized aggregated RBC numbers are plotted with normalized time in Fig. 7c and with the shear rate in Fig. 7d, respectively. As expected, both the normalized aggregated RBC numbers and their variations during a cycle were large at low shear rates and small at high shear rates. RBC behavior under pulsatile flow revealed hysteresis during shear cycling. This phenomenon is attributed to RBCs forming a parabolic rouleaux during the increasing phase of the mean shear rate and the parabolic rouleaux being broken during the following decreasing phase. Therefore, RBC aggregation is significantly related to not only the shear rate value but also its increasing and decreasing phases.

## Discussion

The presentation study performs a numerical investigation to understand in detail the dynamic RBC motion under sinusoidal pulsatile flow conditions. Sinusoidal pulsatile flow has spatial gradients of flow not only in the radial direction but also in the axial direction. This work provides insights into local RBC aggregation which was analyzed with the axial shear rate and its ratio to radial shear rate under sinusoidal pulsatile flow. The formation of local parabolic RBC rouleaux is similar to the previous echogenic variations with a bright collapsing ring (BRCR) observed in a longitudinal view from porcine blood in B-mode images and a human carotid artery in harmonic images^30–34^. The BRCR phenomenon is a bright echogenic ring converging from the tube wall and finally collapsing near the tube center during a pulsatile cycle in the cross-sectional B-mode image. It was hypothesized from the experimental results that flow acceleration is a factor that increases aggregation due to the balance between inertial and compressional forces under pulsatile flow^30^. Experimental approach had limitation to understand distribution of local RBC aggregation under pulsatile flow due to complexity of blood flow in the previous studies. Therefore, we designed a numerical model of RBC kinetics based on interactional and hydrodynamic forces under sinusoidal pulsatile flow. The model in the current paper focused on the relationship between the spatial variations in flow velocity and motion of RBC aggregation under sinusoidal pulsatile flow. To the best of our knowledge, this is the first study to introduce the axial shear rate to explain the local rouleaux pattern under pulsatile flow. It was revealed local parabolic rouleaux formation and its breakage are mainly due to the axial shear rate and its ratio to radial shear rate under pulsatile flow.

Generally, the radial shear rate is greater than the axial shear rate. However, it should be noted that the radial shear rate varies with the radial position of the tube. For example, the radial shear rate is dominant near the tube wall, and the axial shear rate is negligible. At the center of the tube with a low radial shear rate, the axial shear rate has a more important role than the radial shear rate, leading to the parabolic shape of rouleaux and high local hematocrit. Therefore, we determined the variation in the axial shear rate over the radial shear rate at the center of the tube. The simulation results show that the axial shear rate and its ratio to the radial shear rate significantly affect RBC aggregation at the center of the tube. Although the range of the axial shear rate is relatively small (from − 32 ${\mathrm{s}}^{-1}$ to 32 ${\mathrm{s}}^{-1}$) compared with the one of the radial shear rate (from 10 s^−1^ to 100 s^−1^) at the center of the tube, rouleaux formation and its parabolic shape are locally influenced by the axial shear rate.

For verifying various formations and degrees of local RBC aggregation, the number of aggregated RBCs was counted in ROIs. During each step of the simulation, the number of aggregated RBCs was counted and then normalized to the number of non-aggregated RBCs in the initial condition distributed in the corresponding ROIs. A number of aggregated RBCs is a value to quantify the distribution of aggregated RBCs, as shown in Figs. 4, 5, 6 and 7. When the normalized value is greater than one (> 1), the number of RBCs is larger than the number of initial particles (non-aggregated RBCs) in the corresponding ROIs, mainly due to aggregation. For checking the mean aggregate size from the distribution of RBCs, it was confirmed that the normalized aggregated RBC number could represent the local distribution of aggregated RBCs (Fig. 5b). When the normalized number of aggregated RBCs increases, the mean aggregate size also increases, resulting in better observation of parabolic rouleaux. It is a new approach as an indicator to quantify both local aggregation and the degree of RBC aggregation in ROIs. However, this simple counting approach of aggregated RBCs does not sufficiently evaluate the local RBC aggregation and rouleaux patterns separately, requiring further work.

This numerical RBC model simulated the spatial and temporal variations in RBC aggregation under sinusoidal pulsatile flow. The distribution of RBC aggregation was mechanically affected by the interactional and hydrodynamic forces based on Newton's second law under sinusoidal pulsatile flow. In our previous numerical study, the lower the mean flow velocity or the higher the amplitude of the flow velocity is, the clearer the parabolic RBC aggregation and dissociation behavior^26^. Fenech et al*.* reported a numerical RBC model of pulsating shear Couette flow based on Newton's law^25^ to study the microscopic RBC interactions and macroscopic rheological behavior. Hysteresis patterns were found with this model, showing the relationship between the mean aggregation size and triangular pulsating shear rate. From the perspective of the three dynamic forces influencing RBC motion, the interpretation of RBC behavior in that work is similar to that observed in our study, but the rheological sinusoidal flow characteristics affecting the variations in temporal and spatial RBC aggregation were different, resulting in parabolic rouleaux formation in this study.

The mechanisms to understand the hemorheological factors for RBC aggregation were investigated through numerical simulation, but the numerical approach cannot reflect realistic experimental or clinical conditions due to numerical computation the limitations. A quantitative comparison between simulations and experiments was not pursued in this study. It is challenging to provide fundamental morphological properties and blood flow conditions in the simulation due to the limitations of computational time and capability limitations. Biochemical properties such as fibrinogen and polymer were not investigated in terms of their relation to the microscopic mechanism of RBC aggregation, but the interactional forces in the depletion model partially reflect these properties. In the simulation, the significant difference in the RBC suspension in the medium of low-molecular-weight dextran was compared with the effect of increased plasma viscosity and RBC aggregation. It is noted that the distance between two RBCs was the only factor used to determine aggregation in this modeling work.

The interactional and hemodynamic forces principles could be applied to simulate realistic incompressible fluids if the flow velocity profiles showing axial and radial variations could be replaced. In order to satisfy incompressible property of flow, we added an elastic wall condition to the sinusoidal flow profile to simulate the aggregation of RBCs. The supplementary video (see Supplementary video S2 online) shows the parabolic rouleaux under sinusoidal pulsatile flow in an elastic wall that satisfies the volume conservation (top). The red dots represent the mean velocity at the inlet position with time. With the similar condition of mean inlet velocity in a rigid tube, local RBC distribution clearly shows the parabolic rouleaux (bottom). Consequently, the local distribution of RBC aggregation was affected by the axial variation of velocity in both cases. Although the sinusoidal pulsatile flow is artificial, the effect of the axial shear rate on RBC rouleaux is sufficient in reflecting the important role for RBC aggregation.

Indeed, stenosis may provide a velocity profile with a high axial variation as well as radial variation. An elastic vessel wall may also allow the axial variation in flow velocity to be determined by various properties, such as species, site, age, pressure, and behavior of circulatory diseases^35^. Fluid–structure interaction (FSI) simulation results have showed that considerable velocity field variation and wall deformation were caused by high blood pressure under pulsatile flow, which is helps understand the complex interaction of blood flow in an elastic artery^36^. The axial variation in the blood velocity profile in the elastic artery could be simulated in silico and measured in vivo to apply this primary study to future dynamic RBC aggregation studies under pulsatile flow.

## Limitations

### Flow characteristics

In our model, the sinusoidal pulsatile flow was designed to have spatial variation of flow velocity in radial and axial directions. It has the advantages to analyze the spatial variation of velocity field, but it is still unclear whether the realistic arterial flow has similar axial variation of velocity. There are some limitations as follows:

- The flow velocity profile was not derived from the Navier–Stokes equation for incompressible fluid, but was made possible with an assumption of compressible fluid. Sinusoidal pulsatile flow that satisfies the volume conservation with an elastic tube (supplementary video; S2) shows the similar rouleaux pattern where there is axial shear rate in a specific range.
- Due to the limitation of simulation time, this modeling was performed in a micro-channel. It should noted that the flow condition is different from the physiological and realistic conditions, in terms of the morphological properties of blood vessels, pulsation, and asymmetrical motion of the viscoelastic vessel wall. For future studies, simulation will be improved to consider the Womersley flow with an elastic wall for realistic arterial blood flow.
- We did not consider turbulent flow in this simulation.

### Simulation grid and domain

The geometry of flow is a two-dimensional laminar flow in a rigid tube. The tube was composed of a diameter of 0.12 mm and a length of 0.5 mm. The velocity profile and shear rate fields were discretized on collocated grid arrangement. With 100 $\times$ 100 cells of the grid, the uniform grid was generated in the tube. The size of grid was set to be smaller than the radius of a single particle of RBC, and it was designed in consideration of the time step of simulation. It must be noted that, the influence of grid size was not verified in the present study.

### RBC properties

The morphological conditions of physiological characteristics were not considered in this RBC modeling. RBCs were designed to be elastic spheres, and the deformable behavior of RBC was not considered in our simulation.

### Analysis

In this study, the criterion for RBC aggregation was determined by the distance between the two particles. Normalized aggregated RBC number and mean aggregation size (MAS) were used to quantify the local RBC aggregation distribution. A simple approach to count the number of aggregated RBCs in specific ROIs was taken depending on flow velocity or shear rate fields. In the future, we need a new approach to analyze the local RBC aggregation observed in simulation and the BRCR phenomenon in experiments.

## Methods

**Figure 8:**
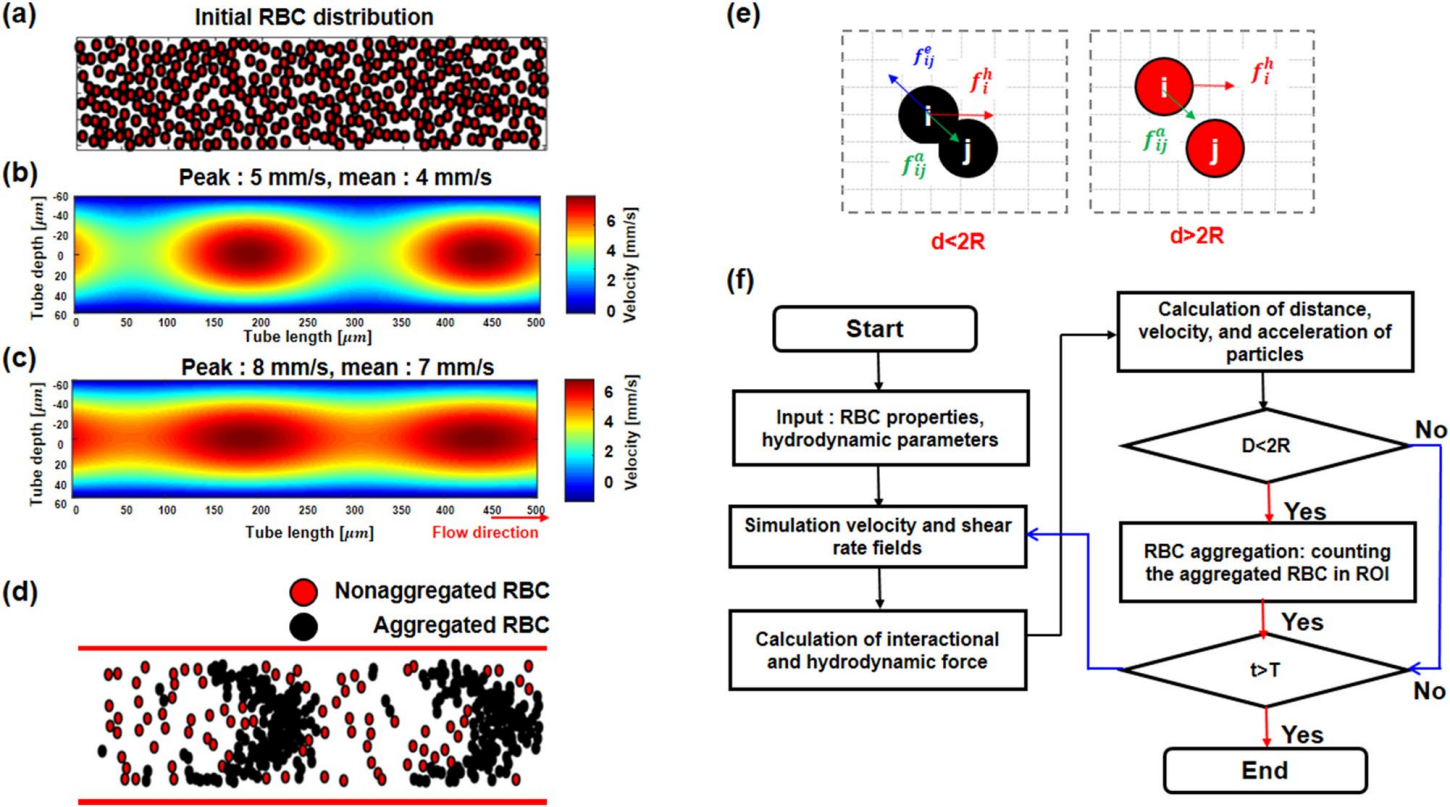
Dynamic RBC modeling and flow chart for analyzing the mechanisms of RBC aggregation under sinusoidal pulsatile flow. (**a**) In the initial step, the particles of 533 RBCs (hematocrits: 40%) were randomly positioned without aggregated RBCs in a 2D rigid tube. (**b**) The flow field represents sinusoidal pulsatile flow with a mean flow velocity of 4 mm/s and a velocity amplitude of 1 mm/s. (**c**) Flow field with a mean flow velocity of 7 mm/s with the same amplitude of velocity. (**d**) Schematic of the RBC distribution in a tube. Red and black particles indicate nonaggregated and aggregated RBCs, respectively. (**e**) Based on the depletion model, the elastic force (${f}_{ij}^{e}$), aggregational force (${f}_{ij}^{a}$), and hydrodynamic force (${f}_{i}^{h}$) determine the distance (*d*), velocity, and acceleration of two particles. *R* is the radius (4 μm) of an RBC. The RBC motion and forces between the particles are calculated during a pulsatile cycle with the designed flow characteristics. (**f**) Flow chart of numerical RBC modeling. In a specific ROI, RBC aggregation was analyzed considering the spatial and temporal variation in radial and axial shear rates under pulsatile flow. *t* is time, and *T* is a period of sinusoidal pulsatile flow. The RBC modeling was implemented using MATLAB (The Math Works, Inc. *MATLAB*. Version 2020a, The Math Works, Inc., 2020. Computer Software. www.mathworks.com).

Numerical simulation was performed to determine the fundamentals of RBC aggregation under sinusoidal pulsatile flow. The overall procedure of the RBC modeling is illustrated in Fig. 8. The visualization and analysis of the motion of RBC particles was implemented using MATLAB software (The Math Works, Inc. *MATLAB*. Version 2020a, The Math Works, Inc., 2020. Computer Software. www.mathworks.com). We focused on the distribution of aggregated RBCs with spatial variation in the flow field in several ROIs. In the numerical simulation, RBCs with a radius of 4 µmwere randomly distributed without overlap in a 2D rectangular rigid tube with dimensions of 0.12 × 0.5 mm^2^ as an initial condition (Fig. 8a). 100 × 100 cells were set in grid, the flow fields had the pixel resolution per single cell with 1.2 $\mathrm{\mu m}$ of height and 5 $\mathrm{\mu m}$ of wide. The size of grid was set smaller than the size of an RBC particle (8 $\mathrm{\mu m}$ of diameter). The spatial discretization was performed on elements with the radial velocity on the vertical cell interfaces, and the axial velocity placed on the horizontal cell interfaces. We numerically set the velocity profile to represent pulsatile flow, which influences the spatial variation in velocity flow fields (amplitude of velocity, mean flow velocity, frequency, etc.). In particular, these properties of flow are hemorheological factors that influence the formation of RBC aggregation and viscosity.

The flow velocity field was controlled by the mean flow velocity from 4 mm/s (Fig. 8b) to 7 mm/s (Fig. 8c) with a fixed velocity amplitude of 1 mm/s. The amplitude was changed to 1.3 mm/s from zero for the Poiseuille flow. We considered a dynamic particle model based on depletion theory for the RBC aggregate mechanism. During sinusoidal cycles, the RBC particles periodically underwent aggregation and exhibited a dissociated distribution of RBC aggregation. The aggregated RBCs and nonaggregated RBCs are shown as black and red particles, respectively (Fig. 8d). In this simulation, during a sinusoidal pulsatile cycle, the distance (${\mathrm{d}}_{i}$), velocity (${\mathrm{v}}_{i}$), and acceleration (${\mathrm{a}}_{i}$) of RBC particle *i* were driven by the three forces of Newton’s second law^25^.

5 $${m}_{i}\frac{{dv}_{i}}{dt}=\sum_{i\ne j}{f}_{ij}+{f}_{i}^{h}$$

6 $${f}_{ij}={f}_{ij}^{a}+{f}_{ij}^{e}$$

The interactional forces, which consist of the elastic force ($${f}_{ij}^{e}),$$ aggregation force ($${f}_{ij}^{a})$$ between RBC particles $$i$$ and $$j$$ and hydrodynamic force ($${f}_{i}^{h}$$), determine whether RBCs form aggregates or not (Fig. 8e). The interactional force between two RBC particles is a function of distance. The elastic force was applied when the distance between two particles was less than the RBC diameter (8 µm). The aggregation force was theoretically calculated based on the depletion model, which was derived from the depletion layer thickness between cell-to-cell distances^24,25,37^. The hydrodynamic force driven by the Stokes drag force was dependent on the relationship between RBC particles and flow conditions at a specific time and position^38,39^. The details of RBC position and motion are described in the Methods section and our previous study^26^. As shown in the flow chart in Fig. 8f, we set the RBC properties, such as radius, mass and random position, viscosity, hematocrit, and the hydrodynamic parameters. The hydrodynamic parameters were changed by changing the amplitude and mean flow velocity. The effect of spatiotemporal variation in the flow velocity field on the RBC aggregation distribution was investigated by counting the number of aggregated RBCs in specific ROIs for a range of hydrodynamic parameters.

*Numerical modeling conditions* In the simulation, the dynamic RBC model was applied to calculate RBC motion based on interactional and hydrodynamic forces to understand RBC aggregation and its spatiotemporal characteristics. The normal shape of RBCs is a biconcave discoid that deforms to pass through microvessels whose diameter is less than the RBC diameter. The deformability of RBCs and RBC shape are important to its physiological and hemorheological roles which are related to RBC aggregation^40^. In RBC modeling, we concentrate on the macroscale aspects of the interrelationship between RBCs based on the interactional and hydrodynamic forces. Therefore, the RBC cross-section is designed to be circular instead of biconcave. The radius and mass of an RBC are 4 µm and 2.94 × 10^−13^ kg, respectively. For initial conditions, 533 RBC-mimicking particles (40% hematocrit) were distributed at random positions without aggregation in a rigid tube with a diameter of 0.12 mm and a length of 0.5 mm. In addition, we set the RBC properties and a flow condition (either steady flow or pulsatile flow) in the model. In our modeling, we imposed periodic boundary conditions in the flow direction due to the observation of RBC aggregate formation in repetitive sinusoidal pulsatile flow with single frequency components. In other words, each particle leaving the end of the tube was repositioned at the entrance with the same acceleration, velocity, radial distance and force vector.

### RBC aggregation mechanism

In the RBC model, the acceleration ($\frac{{dv}_{i}}{dt}$) of particle *i* with respect to time (*t*) determines the inertial force by Newton’s second law. The interactional and hydrodynamic forces determine whether RBCs form aggregates or dissociate. Based on the three forces in Eqs. (7)–(9), RBC motion is governed to show spatial and temporal variation.

RBCs are considered as compressed elastic RBCs in this model. When the distance between the centers of two RBCs is smaller than the cell diameter*,* the elastic force acts on the two particles. The elastic force (${{\varvec{f}}}_{ij}^{e}$) based on a granular interaction model is given by^25^

7 $$ \varvec{f}_{{ij}}^{e}  = \left\{ {\begin{array}{*{20}l}    {k\left( {2R - \varvec{d}_{{\user2{ij}}} } \right)^{{\frac{3}{2}}} n,} \hfill & {if~\varvec{d}_{{\user2{ij}}}  < 2R} \hfill  \\    {0,} \hfill & {otherwise} \hfill  \\   \end{array} } \right. $$

where ${{\varvec{d}}}_{{\varvec{i}}{\varvec{j}}}$ indicates the distance between the centers of mass of two RBCs, *i* and *j*, and *R* is the radius of an RBC particle (*R* = 4 $\mathrm{\mu m}$). The RBCs of a 2-dimensional circle shape are not considered to have deformability and are compressed by the factor modulated by 3 × 10^−6^ N/m of the constant elastic modulus (*k*).

To understand the mechanism of RBC aggregation, we adopt a Morse potential function based on depletion theory. The interaction energy between RBCs in polymer solutions is calculated using the Morse potential energy^23,37^

8 $${\Phi }_{ij}(\delta )={\mathrm{D}}_{e}\left({e}^{2B\left({\delta }_{0}-\delta \right)}-{2e}^{B\left({\delta }_{0}-\delta \right)}\right)$$

9 $${{\varvec{f}}}_{ij}^{a}=2DAB\left({e}^{2B\left({\delta }_{0}-\delta \right)}-{e}^{B\left({\delta }_{0}-\delta \right)}\right){{\varvec{n}}}_{{\varvec{i}}{\varvec{j}}}$$

where *δ* is the cell membrane distance between two RBCs (*δ* = *d* − 2*R*). *δ*_0_ is reference distance. ${\mathrm{D}}_{e}$ and *B* are the surface energy and scaling factor, respectively. *A* indicates the area of the RBC surface in the cell–cell apposition with another cell. From the normalized vector point (${{\varvec{n}}}_{{\varvec{i}}{\varvec{j}}}$) of particles *j* to *i,* the intercellular aggregation force (${{\varvec{f}}}_{ij}^{a}$) is determined by the *DA* and Morse potential functions*. DA* is the energy, which is inversely related to the depletion layer thickness. In this paper, the constant values of *DA, B* and *δ*_0_ are 10^−25^ J, 10^7^ m^−1^ and 11 nm, respectively. The attractive and repulsive forces are determined by the distance between two cells. In the case of flowing fluid, it is known that particle motion dominates the hydrodynamic characteristics. The hydrodynamic properties are not only the viscous properties of the fluid but also the variation of the spatiotemporal distribution of the flow field affecting the movement and mechanism of RBC aggregation. We simply adopted the pseudosteady Stokes drag force^38,39^.

10 $${{\varvec{f}}}_{i}^{h}\left(x,r,t\right)=-6\pi \mu R{U}_{p}(\mathrm{x},\mathrm{r},\mathrm{t})$$

where $$ \overset{\lower0.5em\hbox{$\smash{\scriptscriptstyle\rightharpoonup}$}}{\user2{U}} _{p} \left( {{\text{x}},{\text{r}},{\text{t}}} \right) = ~u\left( {{\text{x}},{\text{r}},{\text{t}}} \right) - ~v_{i} \left( {{\text{x}},{\text{r}},{\text{t}}} \right) $$ is the relative velocity acting on particle *i.* The definition and explanation for the velocity of flow ($$u$$) are given in Eq. (1). $${v}_{i}$$ represents the current particle velocity.

Based on Newton's second law of motion, the acceleration of RBC is proportional to the summation of three forces applied to it. Acceleration, velocity, and position of all particles are computed in each time step. If the distance between center of two particles is less than 8 μm, it is determined that RBCs are aggregated.

*Estimation of RBC aggregation* Counting the RBC aggregates in the rectangular ROI is not sufficient for quantitative analysis because RBC aggregates are affected by temporal and spatial variations in the fluid. Therefore, this simulation was undertaken to provide a basis for the relationship between RBC aggregation distribution and spatial variation in the shear rate's two components. The simulation process was as follows: (1) Shear rate fields were derived by the velocity profile's spatial gradients, including axial and radial directions. (2) RBC aggregation was determined with the shear rate field's spatial distribution by dividing the shear rate field into 10 sections with the same area having a certain range of mean shear rate. In this modeling, the axial to radial shear rate ratio was calculated at the center of the tube (the center width of the tube was set to 16 µm). (3) Finally, the number of aggregated RBCs in each divided section was calculated, with the corresponding spatial distribution of shear rate fields. The number of RBC aggregates was normalized by the number of nonaggregated RBCs with 40% hematocrit at the initial condition in the ROI. This normalized number of RBC aggregates includes information on the degree of aggregation and local hematocrits within the ROI in accordance with the spatial and temporal variation in sinusoidal pulsatile flow. If the normalized number is greater than 1, the local hematocrit is increased mainly due to RBC aggregation in the ROI. However, the normalized number of aggregated RBCs does not directly provide information on the rouleaux size, although it increases with the mean aggregate size on a log scale.

## Supplementary Information

Supplementary Legends.

Supplementary Video 1.

Supplementary Video 2.
