# Supplementary material for: Numerical simulation of spatiotemporal red blood cell aggregation under sinusoidal pulsatile flow
Source: Sci Rep. 2021 May 11;11:9977. doi: 10.1038/s41598-021-89286-1 (PMC8113559; doi:10.1038/s41598-021-89286-1)
Supplement: Supplementary file 1 — Supplementary Legends. [file 41598_2021_89286_MOESM1_ESM.docx]

**Numerical simulation of spatiotemporal red blood cell aggregation under sinusoidal pulsatile flow**

Cheong-Ah Lee ^1^, Dong-Guk Paeng ^1,2,*^

^1^ Department of Ocean System Engineering, Jeju National University, Jeju, Korea
^2^ Department of Radiology and Medical Imaging, University of Virginia, Charlottesville, VA, USA.
^*^[paeng@jejunu.ac.kr](mailto:paeng@jejunu.ac.kr) (corresponding author)

Supplementary material:

S1 : Supplementary video for Figure 2,4

S2 : Supplementary video for discussion and limitation
